# Assessment of Anxiety, Stress, and Depression Among COVID-19 Survivors After 40 Months in the Kurdistan Region of Iraq: An Online Cross-Sectional Study

**DOI:** 10.7759/cureus.63739

**Published:** 2024-07-03

**Authors:** Abdulqader H Hamad, Diyar H Taher, Ahmed A Naif, Iman F Omar, Arpi Manookian, Abdulmalik F Saber, Sirwan K Ahmed, Safin Hussein

**Affiliations:** 1 Department of Psychiatric and Mental Health Nursing, College of Nursing, Hawler Medical University, Erbil, IRQ; 2 Department of Psychiatric and Mental Health, College of Medicine, Hawler Medical University, Erbil, IRQ; 3 Department of Nursing, College of Nursing, Koya Technical Institute, Erbil, IRQ; 4 Department of Medical Surgical Nursing, School of Nursing and Midwifery, Tehran University of Medical Sciences, Tehran, IRN; 5 Department of Nursing, College of Nursing, University of Raparin, Sulaymaniyah, IRQ; 6 Department of Biology, University of Raparin, Sulaymaniyah, IRQ

**Keywords:** covid-19, mental health, depression, stress, anxiety

## Abstract

Background and aims: COVID-19 survivors often experience significant and pervasive psychological distress. This study aimed to investigate the prevalence and demographic factors affecting anxiety, stress, and depression levels among COVID-19 survivors in the Kurdistan region of Iraq.

Method: This online cross-sectional study was conducted from August 1, 2023 to December 17, 2023, in the Kurdistan region of Iraq, including Erbil, Sulaymaniyah, and Duhok. Purposive sampling was used to collect data using an online survey. The survey included demographic information and the Depression, Anxiety, and Stress Scale - 21 Items (DASS-21). Statistical analysis was performed using Stata version 12 (StataCorp LLC, College Station, TX), with frequency and percentage used for categorical variables and mean and standard deviation for quantitative variables. Ordinal regression analyses were conducted to assess associations between demographic factors and mental health outcomes. A p-value of less than 0.05 was considered statistically significant.

Results: A total of 783 participants were enrolled in the study. The mean score for anxiety was 11.62 ± 4.71, indicating moderate levels. For depression, the mean score was 11.54 ± 5.21, indicating mild levels, and for stress, the mean score was 14.0 ± 5.78, indicating normal levels. Younger individuals (15-27) showed higher stress (Estimate: 18.96, P=0.001) and anxiety (Estimate: 2.79, P=0.001) levels compared to older age groups. Males reported significantly lower stress (Estimate: -1.01, P=0.001), anxiety (Estimate: -1.29, P=0.001), and depression (Estimate: -0.72, P=0.001) than females. Participants with a diploma had lower anxiety (Estimate: 0.65, P=0.004) and stress (Estimate: 0.77, P=0.002) levels compared to those with only elementary education.

Conclusions: The study found moderate levels of anxiety among COVID-19 survivors, with mild depression and normal stress levels. To address these issues, it is recommended that policymakers develop targeted mental health interventions. Healthcare providers should focus on early identification and treatment, providing personalized counseling and support to enhance coping mechanisms and overall psychological well-being. By implementing these measures, mental health outcomes for COVID-19 survivors in Iraq can be significantly improved.

## Introduction

The COVID-19 pandemic has left a permanent mark on global health, challenging healthcare systems and imposing a significant burden on individuals' physical and mental well-being [1-3]. In Iraq, as of March 2024, there have been over two million confirmed cases and approximately 25,000 deaths due to COVID-19, placing a substantial strain on the healthcare system [4]. While the physical manifestations of the virus have been extensively documented, the psychological aftermath faced by those who have survived the infection remains an area of critical importance. This is especially evident in the rising cases of anxiety, stress, and depression among COVID-19 survivors. As a result, these mental health concerns have long-lasting implications for their quality of life and ability to reintegrate into society [5]. These conditions can manifest in various forms, including persistent worry, irritability, sleep disturbances, fatigue, and feelings of hopelessness or despair [6]. If left unaddressed, they may contribute to a higher risk of developing chronic mental health disorders, impaired social and occupational functioning, and a diminished overall sense of well-being [7-9].

In this context, the Kurdistan region of Iraq, like many regions globally, has been profoundly impacted by the COVID-19 pandemic, dealing with multiple waves of infections and the subsequent strain on its healthcare infrastructure [10,11]. Despite efforts to alleviate the spread of the virus, the region's population has faced numerous challenges, including disruptions to daily life, economic hardships, and the psychological burden of managing a public health emergency of unparalleled scale [12]. As the region continues to recover from the aftermath of the pandemic, it is essential to understand the psychological impact on those who have survived COVID-19, with a particular focus on the prevalence and severity of anxiety, stress, and depression 40 months post-infection. These mental health conditions can have far-reaching consequences, affecting not only the individuals but also their families, communities, and the overall societal fabric [13].

Thus, examining the psychological well-being of COVID-19 survivors in the Kurdistan region of Iraq is particularly critical given the region's unique socio-cultural context and the potential for long-term mental health implications. The Kurdish population has historically faced numerous challenges, including political instability, economic hardships, and displacement, which may have compounded the psychological impact of the pandemic [14]. Additionally, cultural norms and stigma surrounding mental health issues may hinder individuals from seeking support or accessing appropriate resources [15].

Furthermore, the COVID-19 pandemic has excessively affected certain populations, such as healthcare workers, individuals with pre-existing medical conditions, and those from lower socioeconomic backgrounds [16]. Identifying the specific risk factors and potential disparities in mental health outcomes among COVID-19 survivors in the Kurdistan region is crucial for developing targeted interventions and support systems. By understanding the prevalence and severity of anxiety, stress, and depression among this population, healthcare providers, policymakers, and community organizations can better allocate resources and implement tailored strategies to address these mental health concerns [17].

To address these mental health challenges, various interventions have been explored. One such promising intervention is music therapy. Music therapy has emerged as an effective method for managing depression, anxiety, and stress, particularly in the aftermath of significant health crises such as the COVID-19 pandemic [18]. Research indicates that music therapy can have a profound impact on mental health by reducing symptoms of anxiety and depression, improving mood, and enhancing overall psychological well-being [19]. Through various techniques such as listening to music, songwriting, and engaging in musical activities, individuals can experience emotional release, relaxation, and increased coping mechanisms [20,21]. In the context of COVID-19 survivors, integrating music therapy into mental health support strategies can provide a non-invasive, cost-effective, and culturally adaptable method to alleviate psychological distress and promote recovery. Additionally, cognitive behavioral therapy (CBT) can be effectively used for depression cases that face suicidal ideation and has a significant role in improving mental health outcomes [22-24]. Given its potential benefits, exploring the role of music therapy and CBT in supporting COVID-19 survivors in the Kurdistan region of Iraq is both relevant and timely, offering a complementary approach to traditional mental health interventions.

The findings of this study have the potential to inform public health policies, mental health service provision, and community-based support initiatives aimed at promoting the psychological well-being of COVID-19 survivors in the Kurdistan region of Iraq. Furthermore, this research contributes to the broader understanding of the long-term mental health consequences of the pandemic, informing global efforts to mitigate the psychological impact on those affected. Moreover, this research serves as a critical step toward promoting the overall well-being and resilience of COVID-19 survivors in the Kurdistan region of Iraq, empowering them to navigate the challenges of recovery and reintegration with the necessary psychological support and resources. Therefore, the main aim of this study is to assess the levels of anxiety, stress, and depression 40 months post-infection in people who had COVID-19 in Iraq's Kurdistan region.

## Materials and methods

Study design, setting, and period

This study was an online cross-sectional study conducted in the Kurdistan region of Iraq, specifically in the provinces of Erbil, Sulaymaniyah, and Duhok. Data collection took place from August 1, 2023 to December 17, 2023, using the purposive sampling. The target population was identified through a combination of health records and community outreach efforts. Health records from local health departments provided contact information for COVID-19 survivors, and community outreach involved collaborating with local organizations and leaders to promote the study and encourage participation.

Sample size

To calculate the required sample size for this study, we used the parameters of a 5% margin of error, a 95% confidence interval, and a population proportion of 50%. For an infinite population size, the final sample size would be 385 participants. However, in our survey, we were able to collect more data, totaling 783 cases, thereby enhancing the study's robustness.

Inclusion/exclusion

The study's inclusion criteria were individuals aged 15 or older who had already been diagnosed with COVID-19. Participants needed to be residents of the Kurdistan region of Iraq (specifically Erbil, Sulaymaniyah, and Duhok) and willing to take part in the online survey. Exclusion criteria included individuals with a history of severe psychiatric disorders before contracting COVID-19, those currently experiencing acute medical conditions, and those with long-term illnesses that could predispose them to depression.

Study tools and data collection

The questionnaire was divided into two main parts. The first part gathered demographic data, including age, gender, marital status, educational level, employment status, city of residence, and type of residence. The second part was the Depression, Anxiety, and Stress Scale - 21 Items (DASS-21) [25], which contained 21 items designed to assess levels of depression, anxiety, and stress. To ensure accuracy, the questions were translated from English to Kurdish using a forward-backward method, and the translation was reviewed by psychiatrists to maintain the integrity and accuracy of the translation. In order to guarantee a large-scale distribution and recruitment of participants, the questionnaire was administered in an online format using Google Forms. The survey link was distributed via Facebook, WhatsApp, Twitter, and email lists, targeting Kurdish-speaking groups and pages. Since Kurdistan contains only Erbil, Sulaymaniyah, and Duhok, and the formal language in other parts of Iraq is Arabic, only individuals from Kurdistan, who speak Kurdish, were able to respond. Each participant was allotted a total of 5-10 minutes to complete the questionnaire.

Pilot study

The study questionnaire was tested in an initial pilot study with 25 participants from the general population between March 22 and April 22, 2023, to assess the internal consistency and reliability of the items before they were used in the actual study. The internal consistency of the items was calculated using Cronbach's alpha [26]. For the DASS-21, the overall Cronbach's alpha result came out to be 0.82, indicating acceptable internal consistency and reliability. The data from this initial study were excluded from the final analysis.

Measures

Sociodemographic Characteristics

Several explanatory variables were measured during the study, including age, gender, marital status, educational level, employment status, city of residence, and type of residence.

Depression, Anxiety, and Stress Scale - 21 Items

The second part of the questionnaire was the DASS-21 [25], designed to assess levels of depression, anxiety, and stress. This instrument comprised 21 statements, each rated on a 4-point Likert scale (0 - Did not apply to me at all, 1 - Applied to me to some degree, or some of the time, 2 - Applied to me to a considerable degree or a good part of the time, 3 - Applied to me very much, or most of the time), with total scores ranging across the three subscales. A higher score indicates greater severity of symptoms. The internal consistency of the DASS-21 was assessed using Cronbach's alpha [26], which showed a reliability score of 0.82, indicating acceptable internal consistency.

Ethical approval and informed consent

This study adhered to the guidelines of the Institutional Research Ethics Board and the Declaration of Helsinki. Ethical approval for the study was obtained from Hawler Medical University, College of Nursing, as shown by ethical code 13, granted on 2023-06-11. Informed consent was obtained electronically from all participants before they filled out the online survey. Participants were thoroughly informed about the purpose of the study, the nature of their involvement, and the confidentiality of their responses. Only after acknowledging and agreeing to these terms were participants allowed to proceed with the survey.

Statistical analysis

Data analysis was meticulously conducted, taking into account the nature of the online survey data collection. Descriptive statistics were used to summarize qualitative variables as frequencies and percentages, and quantitative variables as means and standard deviations. Proportions were weighted to reflect the general population, standardized according to WHO population projections for 2000-2025. Ordinal regression analyses were conducted to assess the adjusted associations between key variables of anxiety, stress, depression, and other potential confounding factors. Stata version 12 (StataCorp LLC, College Station, TX) was used for data analysis, with a significance level set at p < 0.05.

## Results

Demographic characteristics

The study's demographic breakdown shows slight female predominance (53.4%) over males (46.6%). Age distribution differs, with most males in the 28-40 age range (60.8%), while females are more evenly spread across 15-27 (30.1%) and 28-40 (40.4%). The average age is 31.82 years, indicating a young adult demographic. A higher proportion of males (71.2%) are married compared to females (62.0%). Educational attainment is similar, with over half holding Bachelor's degrees (males: 54.0%, females: 56.2%), and slightly more females (23.7%) having advanced degrees than males (21.6%). Most participants are self-employed, but more females (13.4%) are employed compared to males (1.1%). Both genders predominantly reside in urban areas (males: 83.3%, females: 88.0%). These demographic insights, detailed in Table 1, contextualize the study's findings and reflect the diverse backgrounds of the participants.

**Table 1:** Demographic characteristics of COVID-19 survivors in the Kurdistan region of Iraq N = 783 represents the total number of participants in the study; SD = Standard Deviation

Demographic Variables	Male %(n)		Female %(n)
Gender	46.6 (365)		53.4 (418)
Age, years			
15-27	27.4 (100)		30.1 (151)
28-40	60.8 (222)		40.4 (203)
41-53	10.1 (37)		20.6 (57)
54-66	1.6 (6)		8.8 (7)
Mean ± SD	31.82 ± 9.01, range 15-66		
Marital status			
Single	28.8 (105)		38.0 (159)
Married	71.2 (260)		62.0 (259)
Education			
Elementary Education	1.4 (5)		0.5 (2)
Secondary Education	8.2 (30)		3.8 (16)
Diploma Degree	14.2 (52)		15.6 (65)
Bachelor's Degree	54.0 (197)		56.2 (235)
Advanced Education	21.6 (79)		23.7 (99)
Illiterate	0.5 (2)		0.2 (1)
Occupation			
Employed	1.1 (4)		13.4 (56)
Self-Employed	85.7 (313)		86.1 (360)
Unemployed	13.2 (48)		0.5 (2)
City			
Erbil	35.6 (130)		30.1 (126)
Sulaymaniyah	33.7 (123)		32.5 (136)
Duhok	30.7 (112)		37.3 (156)
Residence			
Urban	83.3 (304)	88.0 (368)	
Rural	16.7 (61)	12.0 (50)	

Prevalence of anxiety, stress, and depression

According to our findings, the mean and standard deviation for anxiety among participants were 11.62 ± 4.71, indicating moderate anxiety levels. For depression, the mean score was 11.54 ± 5.21, showing mild depression levels. Stress levels were normal, with a mean score of 14.0 ± 5.78. Moreover, the results indicate that a significant proportion of participants fall within the normal range for stress (59.2%) and depression (49.5%). Mild anxiety was experienced by 23.2% of participants. Moderate anxiety was the most prevalent among the three conditions, affecting 32.5% of the participants. Severe levels of anxiety were reported by 23.5% of participants. For stress, 17.3% of participants experienced mild levels, 19.7% experienced moderate levels, and 3.8% experienced severe levels. In terms of depression, 23.9% of participants experienced mild levels, 17.8% experienced moderate levels, and 8.8% experienced severe levels. These findings highlight varying levels of psychological distress among COVID-19 survivors, as detailed in Table 2.

**Table 2:** Distribution of DASS-21 scores by gender in the sample (N = 783)ᵃ DASS, Depression Anxiety Stress Scale; ^a^Values are expressed as No. (%).

Scale	Level	Male (n=365)	Female (n=418)	Total (n=783)
Anxiety Level	Normal	123 (33.7)	40 (9.6)	163 (20.8)
	Mild	100 (27.4)	82 (19.6)	182 (23.2)
	Moderate	88 (24.1)	167 (40.0)	255 (32.5)
	Severe	54 (14.8)	129 (30.9)	183 (23.5)
	Mean ± SD	11.62 ± 4.71	-	-
Stress Level	Normal	264 (72.3)	200 (47.8)	464 (59.2)
	Mild	53 (14.5)	82 (19.6)	135 (17.3)
	Moderate	41 (11.2)	113 (27.0)	154 (19.7)
	Severe	7 (1.9)	23 (5.5)	30 (3.8)
	Mean ± SD	14.0 ± 5.78	-	-
Depression Level	Normal	215 (58.9)	172 (41.1)	387 (49.5)
	Mild	80 (21.9)	107 (25.6)	187 (23.9)
	Moderate	52 (14.2)	88 (21.1)	140 (17.8)
	Severe	18 (4.9)	51 (12.2)	69 (8.8)
	Mean ± SD	11.54 ± 5.21	-	-

Demographic factors affecting on depression, anxiety, and stress levels

Table 3 reveals several significant factors affecting stress, anxiety, and depression levels among participants. Younger individuals (15-27) showed higher stress (Estimate: 18.96, P=0.001) and anxiety (Estimate: 2.79, P=0.001) levels compared to older age groups. Males reported significantly lower stress (Estimate: -1.01, P=0.001), anxiety (Estimate: -1.29, P=0.001), and depression (Estimate: -0.72, P=0.001) than females. Participants with a diploma had lower anxiety (Estimate: 0.65, P=0.004) and stress (Estimate: 0.77, P=0.002) levels compared to those with only elementary education. Employed individuals experienced higher depression levels (Estimate: 0.89, P=0.03) than unemployed ones. Residents of Erbil reported higher stress (Estimate: 18.37, P=0.001), anxiety (Estimate: 2.35, P=0.001), and depression (Estimate: 2.07, P=0.003) than those in Duhok, while Sulaymaniyah residents also reported higher stress (Estimate: 17.97, P=0.001) and anxiety (Estimate: 2.01, P=0.001). Marital status, residence type, and occupation were not significantly associated with mental health outcomes.

**Table 3:** Ordinal regression of factors affecting DASS-21 scores in COVID-19 survivors (Link = Logit)ᵃ Statistical test: Ordinal regression analysis. Significance was set at P < 0.05. LB, lower bound; UB, upper bound; CI, Confidence Interval; DASS, Depression, Anxiety, and Stress Scale. ^a^ Reference parameter

Variables	Stress				Anxiety				Depression			
	Estimate	%95 CI		P-value	Estimate	%95 CI		P-value	Estimate	%95 CI		P-value
		LB	UB			LB	UB			LB	UB	
Age												
15-27	18.96	17.59	20.34	0.001	2.79	1.27	4.32	0.001	1.38	-0.36	3.12	0.12
28-40	1.26	-0.07	2.58	0.06	0.92	-0.19	2.02	0.10	0.11	-0.99	1.22	0.84
41-53	0.48	-0.87	1.83	0.49	0.27	-0.85	1.39	0.64	-0.30	-1.43	0.83	0.60
54-66	0 ^a^	-	-	-	0 ^a^	-	-	-	0 ^a^	-	-	-
Gender												
Male	-1.01	-1.42	-0.78	0.001	-1.29	-1.57	-0.10	0.001	-0.72	-1.01	-0.42	0.001
Female	0 ^a^	-	-	-	0 ^a^	-	-	-	0 ^a^	-	-	-
Marital Status												
Married	-0.13	-0.48	0.22	0.48	-0.10	-0.42	0.22	0.54	-0.21	-1.01	-0.42	0.21
Single	0 ^a^	-	-	-	0 ^a^	-	-	-	0 ^a^	-	-	-
Education												
Elementary Education	0.87	-0.74	2.48	0.29	0.05	-1.31	1.49	0.95	-0.19	-1.76	1.37	0.81
Secondary Education	0.35	-0.40	1.10	0.36	-0.52	-1.17	0.14	0.12	-0.08	-0.77	0.62	0.83
Diploma Degree	0.77	0.29	1.26	0.002	0.65	0.21	1.09	0.004	0.48	0.03	0.92	0.04
Bachelor's Degree	0.47	0.09	0.85	0.02	0.10	-0.23	0.43	0.56	-0.07	-0.41	0.27	0.69
Advanced Education	1.95	-0.20	4.11	0.08	21.42	21.41	21.42	0.53	2.74	0.38	5.10	0.02
Illiterate	0 ^a^	-	-	-	0 ^a^	-	-	-	0 ^a^	-	-	-
Occupation												
Employed	0.75	-0.09	1.59	0.08	0.20	-0.54	0.95	0.60	0.89	0.10	1.67	0.03
Self-Employed	0.07	-0.64	0.79	0.84	-0.12	-0.71	0.48	0.70	0.18	-0.47	0.83	0.59
Unemployed	0 ^a^	-	-	-	0 ^a^	-	-	-	0 ^a^	-	-	-
City												
Erbil	18.37	17.97	18.77	0.001	2.35	1.27	3.43	0.001	2.07	0.71	3.43	0.003
Sulaymaniyah	17.97	17.97	17.98	0.001	2.01	0.96	3.07	0.001	1.81	0.47	3.16	0.008
Duhok	0 ^a^	-	-	-	0 ^a^	-	-	-	0 ^a^	-	-	-
Residence Type												
Urban	-0.09	-0.50	0.33	0.69	-0.11	-0.57	0.18	0.31	-0.25	-0.64	0.13	0.11
Rural	0 ^a^	-	-	-	0 ^a^	-	-	-	0 ^a^	-	-	-

## Discussion

The present study aimed to assess the prevalence of anxiety, stress, and depression among COVID-19 survivors in the Kurdistan region of Iraq, approximately 40 months after the initial outbreak. Understanding the long-term mental health impacts of COVID-19 is crucial for developing appropriate interventions and supporting the well-being of survivors. Overall, the results revealed that the participants experienced moderate levels of anxiety, mild depression, and normal stress. The findings shed light on the varying levels of psychological distress experienced by this population and the potential influencing factors.

The COVID-19 pandemic has had a profound impact on global mental health, with numerous studies documenting increased rates of anxiety, depression, and other psychological disorders [27,28]. While the initial focus was on the acute mental health consequences during the peak of the pandemic, there is a growing recognition of the need to understand the long-term effects on individuals who have recovered from COVID-19. As the pandemic continues to evolve, it is essential to explore how survivors cope with lingering psychological distress and to identify the factors that influence their mental well-being over time. Consequently, the researchers chose this topic to provide insights into the long-term mental health impacts of COVID-19 on survivors in the Kurdistan region of Iraq, to inform the development of targeted interventions to support their well-being in Kurdish society.

The study's demographic breakdown revealed a slight female predominance, aligning with previous research suggesting that women may be more susceptible to mental health issues during and after the pandemic [29]. The age distribution, skewed towards younger adults, is consistent with the higher risk of psychological distress observed in this age group during the COVID-19 pandemic [30]. The diverse educational and socioeconomic backgrounds of the participants provide valuable insights into the potential influence of these factors on mental health outcomes. This demographic insight is crucial as it helps contextualize the varying levels of anxiety, stress, and depression observed in the study, reflecting the broader trends seen globally.

The prevalence data highlighted the varying levels of psychological distress among COVID-19 survivors, with a significant proportion experiencing mild to moderate anxiety. These findings are consistent with previous studies reporting elevated anxiety levels in individuals who have recovered from COVID-19 [31,32]. The persistence of anxiety symptoms post-recovery may be attributed to the lingering impact of the illness, fear of recurrence, or residual physiological and psychological effects [33]. Such ongoing anxiety underscores the need for continued mental health support and interventions tailored to the specific experiences of COVID-19 survivors. Addressing these anxiety levels is crucial for improving the overall well-being and quality of life for those who have endured the virus.

The higher levels of stress and anxiety observed in younger individuals align with existing literature suggesting that younger age groups may be more vulnerable to mental health challenges during and after the pandemic [30]. This vulnerability could be related to factors such as disruptions in education and career trajectories, social isolation, and financial instability, which disproportionately impact younger populations [34]. These findings highlight the need for targeted mental health interventions for younger individuals to address these specific stressors and support their psychological well-being. Implementing such tailored approaches can help mitigate the long-term mental health impacts on this particularly affected demographic

The gender differences observed in the study, with males reporting significantly lower levels of stress, anxiety, and depression than females, are supported by previous research that has consistently documented higher rates of mental health issues among women during the COVID-19 pandemic [35,36]. These differences may be attributable to sociocultural factors, coping mechanisms, and gender-based disparities in the impact of the pandemic on various aspects of life. The protective effect of higher educational attainment on psychological distress, evidenced by the lower levels of anxiety and stress among participants with a diploma compared to those with only elementary education, aligns with existing literature [37]. Education can influence an individual's ability to access and understand health information, adopt effective coping strategies, and navigate challenging circumstances, thereby mitigating the impact of stressors on mental health.

The finding that employed individuals experience higher levels of depression compared to unemployed ones is noteworthy and may be attributable to occupational stressors, work-life balance challenges, and the potential impact of COVID-19 on job security and financial stability [38]. However, it is important to consider potential confounding factors such as income level and job satisfaction, which could influence the relationship between employment status and mental health outcomes. The geographical differences observed in the study, with residents of Erbil and Sulaymaniyah reporting higher levels of stress, anxiety, and depression compared to those in Duhok, highlight the importance of considering regional factors in the mental health outcomes of COVID-19 survivors. These variations may be influenced by factors such as healthcare infrastructure, sociocultural norms, and the specific impact of the pandemic on different regions [39].

Interestingly, the study did not find significant associations between marital status, residence type, and occupation with mental health outcomes, suggesting that these factors may not play a crucial role in determining the psychological distress experienced by COVID-19 survivors in this context. However, it is essential to interpret these findings with caution, as the interplay of various sociodemographic and cultural factors can be complex and may differ across different populations and contexts. These results underscore the need for a nuanced understanding of mental health determinants, taking into account the unique circumstances and experiences of different demographic groups.

Limitations and directions for future research

One key limitation of the study is that its findings are specific to the Kurdistan region of Iraq and may not apply to other regions of Iraq or other populations. Future research should focus on conducting similar studies in different regions of Iraq and among diverse populations to enhance the generalizability of the findings. Additionally, further studies could explore the long-term effects of various interventions, such as music therapy and CBT, on mental health outcomes in COVID-19 survivors.

## Conclusions

The study revealed that the participants experienced moderate levels of anxiety, along with mild depression and normal stress. In order to tackle these issues, policymakers are advised to develop targeted mental health interventions that specifically address anxiety and depression in individuals who have survived COVID-19. These interventions may involve improving access to mental health services, incorporating mental health screenings into regular healthcare visits, and establishing community-based support programs. Healthcare providers must prioritize the early detection and treatment of anxiety and depression, offering personalized counseling and support to enhance patients' coping skills and overall psychological well-being. By implementing these measures, the mental health outcomes for COVID-19 survivors in Iraq can be significantly enhanced. To advance knowledge in this field, interdisciplinary collaboration is essential. Integrating insights from neuroscience, psychology, and musicology can foster innovative approaches to mental healthcare. By combining these disciplines, we can develop more effective, holistic interventions that address the complex needs of COVID-19 survivors. Additionally, practical implications for clinicians and researchers include the development of personalized music interventions and standardized protocols to ensure consistent and effective treatment outcomes.

## Appendices

Questionnaire

This questionnaire is integral to our article titled " Assessment of Anxiety, Stress, and Depression Among COVID-19 Survivors 40 Months After in the Kurdistan Region of Iraq: An Online Cross-Sectional Study." Your participation is entirely voluntary and highly valued. The questionnaire guarantees complete anonymity; hence, please do not provide any personal identifiers such as your name. Your insights are important to this study, and I am grateful for the time you dedicate to completing this survey.

Code .....…

Name of the City ………………………

**Table 4:** Sociodemographic characteristics questionnaire Respondents are asked to tick the option that fits them for each category.

Part 1: Sociodemographic Characteristics	Options	Response
X1: Age (Years)		
X2: Gender	1. Male	
	2. Female	
X3: Education	1. Elementary	
	2. Secondary	
	3. Diploma	
	4. Bachelor	
	5. Advanced Education	
	6. Illiterate	
X4: Occupation	1. Employed	
	2. Self-Employed	
	3. Unemployed	
X5: Residence	1. Urban	
	2. Rural	

Part two: Depression, Anxiety and Stress Scale - 21 items (DASS-21)

Please read each statement and circle a number 0, 1, 2 or 3 which indicates how much the statement applied to you over the past week. There are no right or wrong answers. Do not spend too much time on any statement.

**Table 5:** Depression, Anxiety, and Stress Scale - 21 Items (DASS-21) Respondents are asked to rate each statement based on how much it applied to them over the past week. The rating scale is as follows: 0 - Did not apply to me at all, 1 - Applied to me to some degree, or some of the time, 2 - Applied to me to a considerable degree, or a good part of the time, 3 - Applied to me very much, or most of the time. The letters in the parentheses [(s), (a), (d)] signify the subscales of the DASS-21: (s) Stress, (a) Anxiety, (d) Depression

Item No.	Statement	0	1	2	3
1 (s)	I found it hard to wind down	0	1	2	3
2 (a)	I was aware of dryness of my mouth	0	1	2	3
3 (d)	I couldn’t seem to experience any positive feeling at all	0	1	2	3
4 (a)	I experienced breathing difficulty (e.g. excessively rapid breathing, breathlessness in the absence of physical exertion)	0	1	2	3
5 (d)	I found it difficult to work up the initiative to do things	0	1	2	3
6 (s)	I tended to over-react to situations	0	1	2	3
7 (a)	I experienced trembling (e.g. in the hands)	0	1	2	3
8 (s)	I felt that I was using a lot of nervous energy	0	1	2	3
9 (a)	I was worried about situations in which I might panic and make a fool of myself	0	1	2	3
10 (d)	I felt that I had nothing to look forward to	0	1	2	3
11 (s)	I found myself getting agitated	0	1	2	3
12 (s)	I found it difficult to relax	0	1	2	3
13 (d)	I felt down-hearted and blue	0	1	2	3
14 (s)	I was intolerant of anything that kept me from getting on with what I was doing	0	1	2	3
15 (a)	I felt I was close to panic	0	1	2	3
16 (d)	I was unable to become enthusiastic about anything	0	1	2	3
17 (d)	I felt I wasn’t worth much as a person	0	1	2	3
18 (s)	I felt that I was rather touchy	0	1	2	3
19 (a)	I was aware of the action of my heart in the absence of physical exertion (e.g. sense of heart rate increase, heart missing a beat)	0	1	2	3
20 (a)	I felt scared without any good reason	0	1	2	3
21 (d)	I felt that life was meaningless	0	1	2	3
